# Supplementary material for: Copy number amplification of ENSA promotes the progression of triple-negative breast cancer via cholesterol biosynthesis
Source: Nat Commun. 2022 Feb 10;13:791. doi: 10.1038/s41467-022-28452-z (PMC8831589; doi:10.1038/s41467-022-28452-z)

Supplementary Table 1. Characteristics of the organoids

ID	Gender	Type	Grade	T (cm)	LN pos	LN	ER	PR	HER2	KI67	LVI
1	Female	IDC	III	2.6	0	28	-	-	-	80%	+
2	Female	IDC	III	5.5	25	27	-	-	-	70%	+
3	Female	IDC	III	2.5	0	4	-	-	-	35%	-

IDC: invasive ductal carcinoma; T: tumor size; LN pos: the number of positive lymph nodes; LN: the number of total lymph nodes measured; ER: estrogen receptor; PR: progestogen receptor; HER2: human epidermal growth factor receptor 2; LVI: lymphovascular invasion

Supplementary Table 2. Clinicopathological features of the mini-PDXs

Sample ID	Gender	TNM	Grade	Histopathologic type	ER	PR	HER2
543	Female	T2N0M0	II	IDC	-	-	-
552	Female	T2N0M0	II	IDC	-	-	-
553	Female	T2N0M0	III	IDC	-	-	-
554	Female	T1N0M0	III	IDC	-	-	-
573	Female	T2N1M0	III	IDC	-	-	-
578	Female	T1N0M0	II	IDC	-	-	-
584	Female	T1N1M0	III	IDC	-	-	-

T: tumor size; N: lymph node; M: metastasis; IDC: invasive ductal carcinoma; ER: estrogen receptor; PR: progestogen receptor; HER2: human epidermal growth factor receptor 2.

Supplementary Table 3. Multivariate cox regression analysis of RFS in 138 primary TNBC

	Univariate Cox		Multivariate Cox	
	HR (95% CI)	*P* value	HR (95% CI)	*P* value
ENSA IHC score<br>High vs. Low	2.4<br>(1.17-4.95)	0.02	2.27<br>(1.06-4.86)	0.04
Age<br>≥50y vs. ＜50y	0.41<br>(0.20-0.84)	0.01	0.51<br>(0.24-1.09)	0.08
Tumor size<br>≥2cm vs. ＜2cm	0.95<br>(0.38-2.36)	0.9	0.77<br>(0.31-1.92)	0.57
Lymph node<br>N1-3 vs. N0	2.16<br>(1.04-4.49)	0.04	2.28<br>(1.06-4.91)	0.03

RFS: relapse-free survival; TNBC: triple-negative breast cancer; HR: hazard ratio; CI: confidence intervals; IHC: immunohistochemistry

Supplementary Table 4. Multivariate cox regression analysis of OS in 138 primary TNBC

	Univariate Cox		Multivariate Cox	
	HR (95% CI)	*P* value	HR (95% CI)	*P* value
ENSA IHC score<br>High vs. Low	4.1<br>(1.16-14.56)	0.03	1.36<br>(0.97-1.91)	0.07
Age<br>≥50y vs. ＜50y	0.24<br>(0.06-0.92)	0.04	0.34<br>(0.08-1.41)	0.13
Tumor size<br>≥2cm vs. ＜2cm	2.42<br>(0.30-19.33)	0.41	1.59<br>(0.19-12.96)	0.66
Lymph node<br>N1-3 vs. N0	3.25<br>(0.84-12.58)	0.09	2.78<br>(0.69-11.24)	0.15

OS: overall survival; TNBC: triple-negative breast cancer; HR: hazard ratio; CI: confidence intervals; IHC: immunohistochemistry

Supplementary Table 5. A list of primers for RT-qPCR

ENSA-Forward	AGGCAAAGCTAAAGGCCAAAT
ENSA-Reverse	GCCATGTTGTAGTCTCCTGAGT
FDPS-Forward	TGTGACCGGCAAAATTGGC
FDPS-Reverse	GCCCGTTGCAGACACTGAA
DHCR7-Forward	GCTGCAAAATCGCAACCCAA
DHCR7-Reverse	GCTCGCCAGTGAAAACCAGT
FDFT1-Forward	CCACCCCGAAGAGTTCTACAA
FDFT1-Reverse	TGCGACTGGTCTGATTGAGATA
ACAT2-Forward	GCGGACCATCATAGGTTCCTT
ACAT2-Reverse	ACTGGCTTGTCTAACAGGATTCT
HMGCR-Forward	TGATTGACCTTTCCAGAGCAAG
HMGCR-Reverse	CTAAAATTGCCATTCCACGAGC
EBP-Forward	CTCAGCACCTAAGACTGGACA
EBP-Reverse	ACGACTAAGACCCCTGTGACA
MVD-Forward	CTCCCTGAGCGTCACTCTG
MVD -Reverse	GGTCCTCGGTGAAGTCCTTG
MVK -Forward	CATGGCAAGGTAGCACTGG
MVK -Reverse	GATACCAATGTTGGGTAAGCTGA
LSS-Forward	GCACTGGACGGGTGATTATGG
LSS-Reverse	TCTCTTCTCTGTATCCGGCTG
SREBF2-Forward	AACGGTCATTCACCCAGGTC
SREBF2-Reverse	GGCTGAAGAATAGGAGTTGCC
GAPDH-Forward	GGAGCGAGATCCCTCCAAAAT
GAPDH-Reverse	GGCTGTTGTCATACTTCTCATGG
HMGCS1-Forward	GATGTGGGAATTGTTGCCCTT
HMGCS1-Reverse	ATTGTCTCTGTTCCAACTTCCAG

RT-qPCR: real-time quantitative reverse transcription.

Supplementary Table 6. Detailed antibody usage

Name	Company	Application	Dilution	Cat #
ENSA	Abcam	Immunoblot	1:10000	ab180513
ENSA	Proteintech	IHC	1:100	14518-1-AP
MVK	Affinity	Immunoblot	1:1000	DF3238
FDPS	Proteintech	Immunoblot; IHC	WB: 1:3000; IHC: 1:200	16129-1-AP
ACAT2	Abcam	Immunoblot	1:3000	ab131215
SREBP2	Abcam	Immunoblot; IHC	WB: 1:1000; IHC: 1:100	ab30682
SREBP2	Proteintech	IHC	1:200	28212-1-AP
LSS	Abcam	Immunoblot	1:3000	ab140124
FDFT1	Abcam	Immunoblot	1:3000	ab195046
HMGCR	Abcam	Immunoblot	1:3000	ab174830
HMGCS1	Proteintech	Immunoblot	1:1000	17643-1-AP
FLAG	Sigma-Aldrich	Immunoblot	1:1000	F1804
p-STAT3 (Tyr705)	Cell Signaling	Immunoblot; IHC	1:2000	9145
p-STAT3 (Ser 727)	Cell Signaling	Immunoblot	1:1000	9134
STAT3	Cell Signaling	Immunoblot; ChIP	WB: 1:1000; ChIP: 4μg	9139
PP2AC	Cell Signaling	Immunoblot	1:2000	2038
GAPDH	Proteintech	Immunoblot	1:10000	60004-1-Ig
alpha tubulin	Proteintech	Immunoblot	1:3000	11224-1-AP
mouse IgG2a isotype	Cell Signaling	ChIP	4ug	61656
HRP-linked anti-mouse antibodies	Proteintech	Immunoblot	1:5000	SA00001-1
HRP-linked anti-rabbit antibodies	Proteintech	Immunoblot	1:5000	SA00001-2

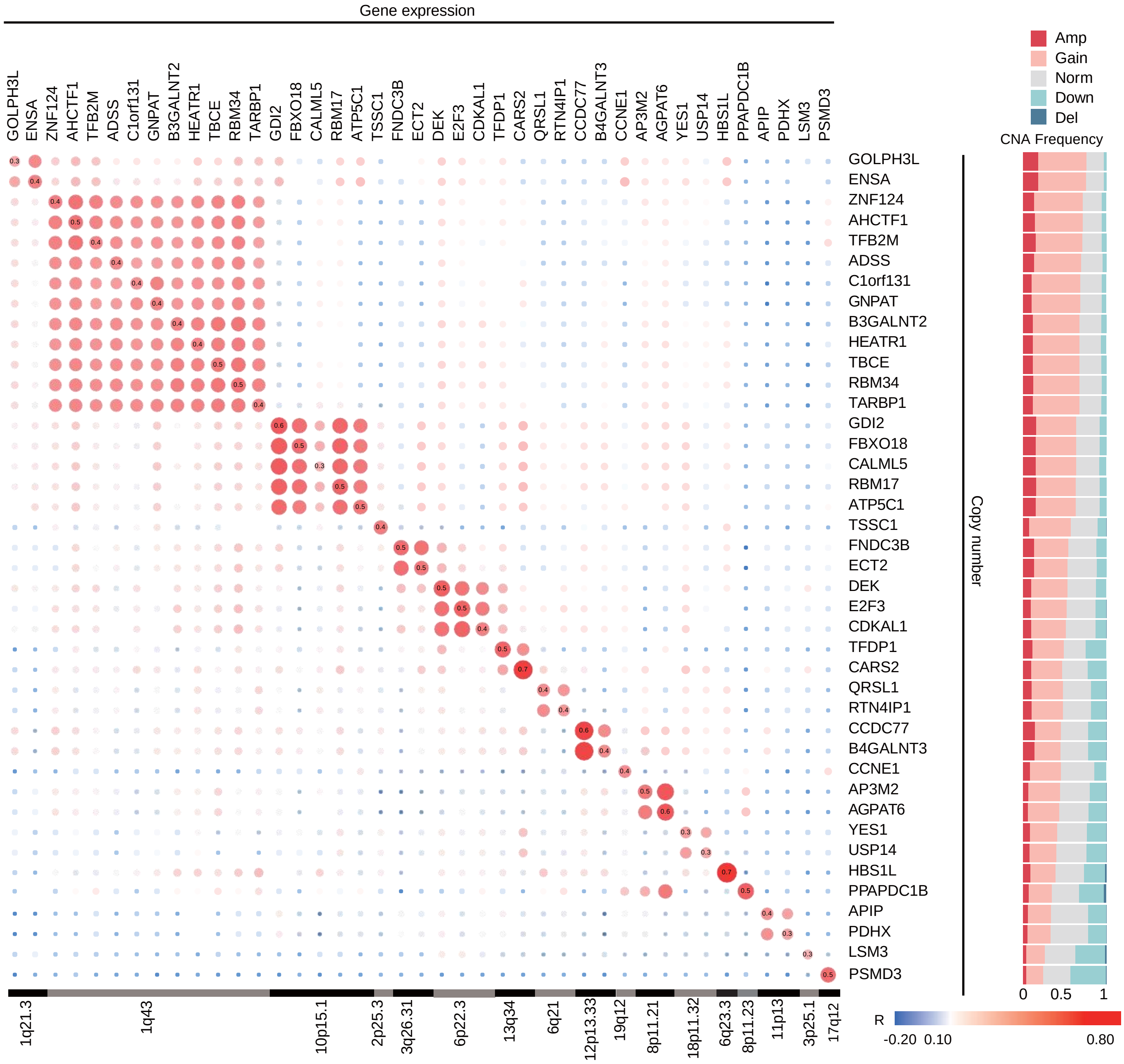

Gene expression
GOLPH3L
ENSA
ZNF124
AHCTF1
TFB2M
ADSS
C1orf131
GNPAT
B3GALNT2
HEATR1
TBCE
RBM34
TARBP1
GDI2
FBXO18
CALML5
RBM17
ATP5C1
TSSC1
FNDC3B
ECT2
DEK
E2F3
CDKAL1
TFDP1
CARS2
QRSL1
RTN4IP1
CCDC77
B4GALNT3
CCNE1
AP3M2
AGPAT6
YES1
USP14
HBS1L
PPAPDC1B
APIP
PDHX
LSM3
PSMD3
Copy number
Amp
Gain
Norm
Down
Del
CNA Frequency
0
0.5
1
1q21.3
1q43
10p15.1
2p25.3
3q26.31
6p22.3
13q34
6q21
12p13.33
19q12
8p11.21
18p11.32
6q23.3
8p11.23
11p13
3p25.1
17q12
R
-0.20
0.10
0.80

Supplementary Fig. 1 The correlation between the expression and copy number of 41 candidates in 302 TNBC patients according to both RNA-seq data and copy number data. Genes were ranked by amplification and gain frequency. Correlation coefficients were calculated using the Pearson test. Amp, amplification; Norm, normal; Del, deletion.

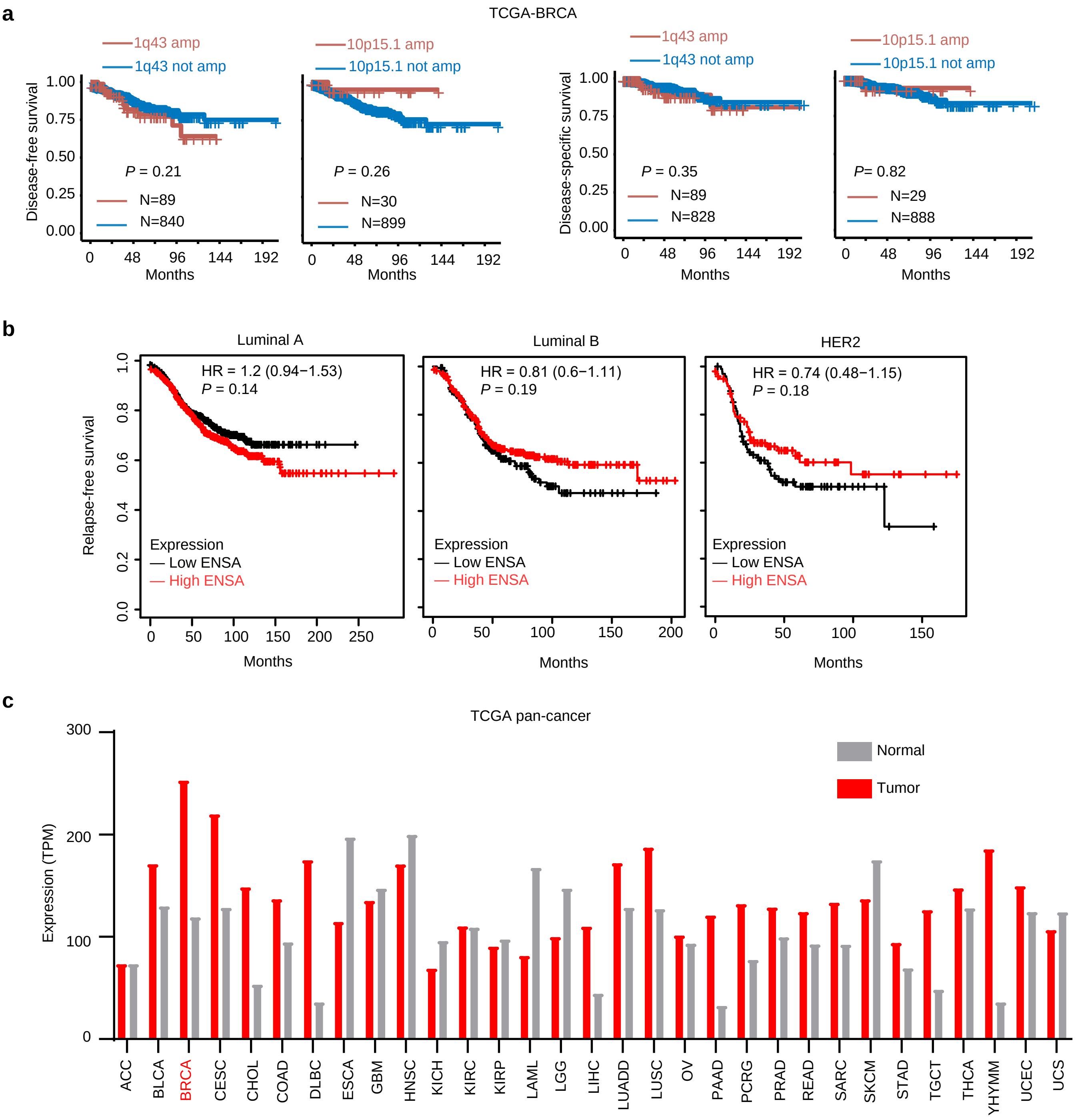
a
TCGA-BRCA
1q43 amp
1q43 not amp
Disease-free survival
1.00
0.75
0.50
0.25
0.00
P = 0.21
N=89
N=840
0
48
96
144
192
Months
10p15.1 amp
10p15.1 not amp
P = 0.26
N=30
N=899
Months
1q43 amp
1q43 not amp
Disease-specific survival
P = 0.35
N=89
N=828
Months
10p15.1 amp
10p15.1 not amp
P= 0.82
N=29
N=888
Months
b
Luminal A
Relapse-free survival
1.0
0.8
0.6
0.4
0.2
0.0
HR = 1.2 (0.94−1.53)
P = 0.14
Expression
— Low ENSA
— High ENSA
0
50
100
150
200
250
Months
Luminal B
HR = 0.81 (0.6−1.11)
P = 0.19
Expression
— Low ENSA
— High ENSA
Months
HER2
HR = 0.74 (0.48−1.15)
P = 0.18
Expression
— Low ENSA
— High ENSA
Months
c
TCGA pan-cancer
Expression (TPM)
300
200
100
0
Normal
Tumor
ACC
BLCA
BRCA
CESC
CHOL
COAD
DLBC
ESCA
GBM
HNSC
KICH
KIRC
KIRP
LAML
LGG
LIHC
LUADD
LUSC
OV
PAAD
PCRG
PRAD
READ
SARC
SKCM
STAD
TGCT
THCA
YHYMM
UCEC
UCS

Supplementary Fig. 2 ENSA transcriptome profile in breast cancer. (**a**) Kaplan-Meier survival analysis of 1q43 and 10p15.1 copy number alteration in TCGA breast cancer patients. Log-rank test. (**b**) Kaplan-Meier plots of ENSA in the luminal A, luminal B and HER2 subtypes (https://kmplot.com/analysis/). Log-rank test. (**c**) ENSA mRNA levels in different cancers from the TCGA cohort. TCGA, The Cancer Genome Atlas; BRCA, breast cancer; amp, amplification. TPM, transcripts per million.

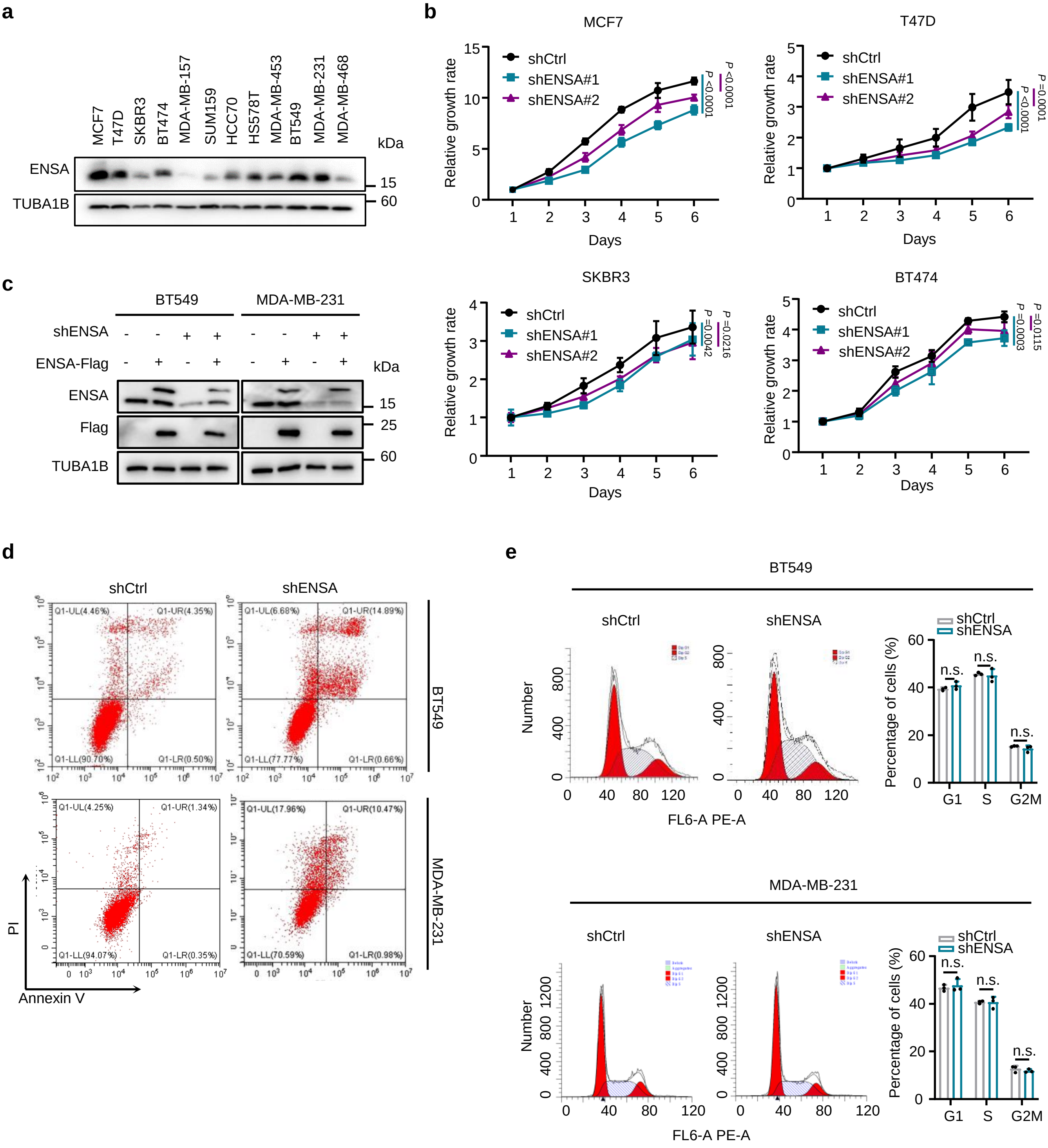

a
MCF7
T47D
SKBR3
BT474
MDA-MB-157
SUM159
HCC70
HS578T
MDA-MB-453
BT549
MDA-MB-231
MDA-MB-468
kDa
ENSA
15
TUBA1B
60
b
MCF7
T47D
SKBR3
BT474
shCtrl
shENSA#1
shENSA#2
Relative growth rate
Days
P <0.0001
P <0.0001
P =0.0001
P <0.0001
P =0.0216
P =0.0042
P =0.0115
P =0.0003
c
BT549
MDA-MB-231
shENSA
ENSA-Flag
kDa
ENSA
Flag
TUBA1B
15
25
60
d
shCtrl
shENSA
BT549
MDA-MB-231
PI
Annexin V
Q1-UL(4.46%)
Q1-UR(4.35%)
Q1-LL(90.70%)
Q1-LR(0.50%)
Q1-UL(6.68%)
Q1-UR(14.89%)
Q1-LL(77.77%)
Q1-LR(0.66%)
Q1-UL(4.25%)
Q1-UR(1.34%)
Q1-LL(94.07%)
Q1-LR(0.35%)
Q1-UL(17.96%)
Q1-UR(10.47%)
Q1-LL(70.59%)
Q1-LR(0.98%)
e
BT549
MDA-MB-231
shCtrl
shENSA
Number
FL6-A PE-A
Percentage of cells (%)
n.s.
G1
S
G2M

Supplementary Fig. 3 ENSA promotes the survival of TNBC cells. (a) The protein expression of ENSA across multiple breast cancer cell lines. **(b)** In vitro growth curves of MCF7, T47D, SKBR3, and BT474 cells expressing control or ENSA shRNA. n=6. Data are presented as mean ± SD. Two-tailed two-way ANOVA tests. **(c)** Empty vector or flag-tagged ENSA overexpression vector was transfected into BT549 and MDA-MB-231 cells expressing control or ENSA shRNA. **(d)** Representative images of cell apoptosis in BT549 and MDA-MB-231 cells expressing control or ENSA shRNA. **(e)** Cell cycle distribution measured in BT549 and MDA-MB-231 cells expressing control or ENSA shRNA. Representative images and the percentage of cells in the G1/S/G2M phase are shown. n=3. Data are presented as mean ± SD. Two-tailed unpaired Student's t tests. Source data are provided as a Source Data file. n.s., not significant.

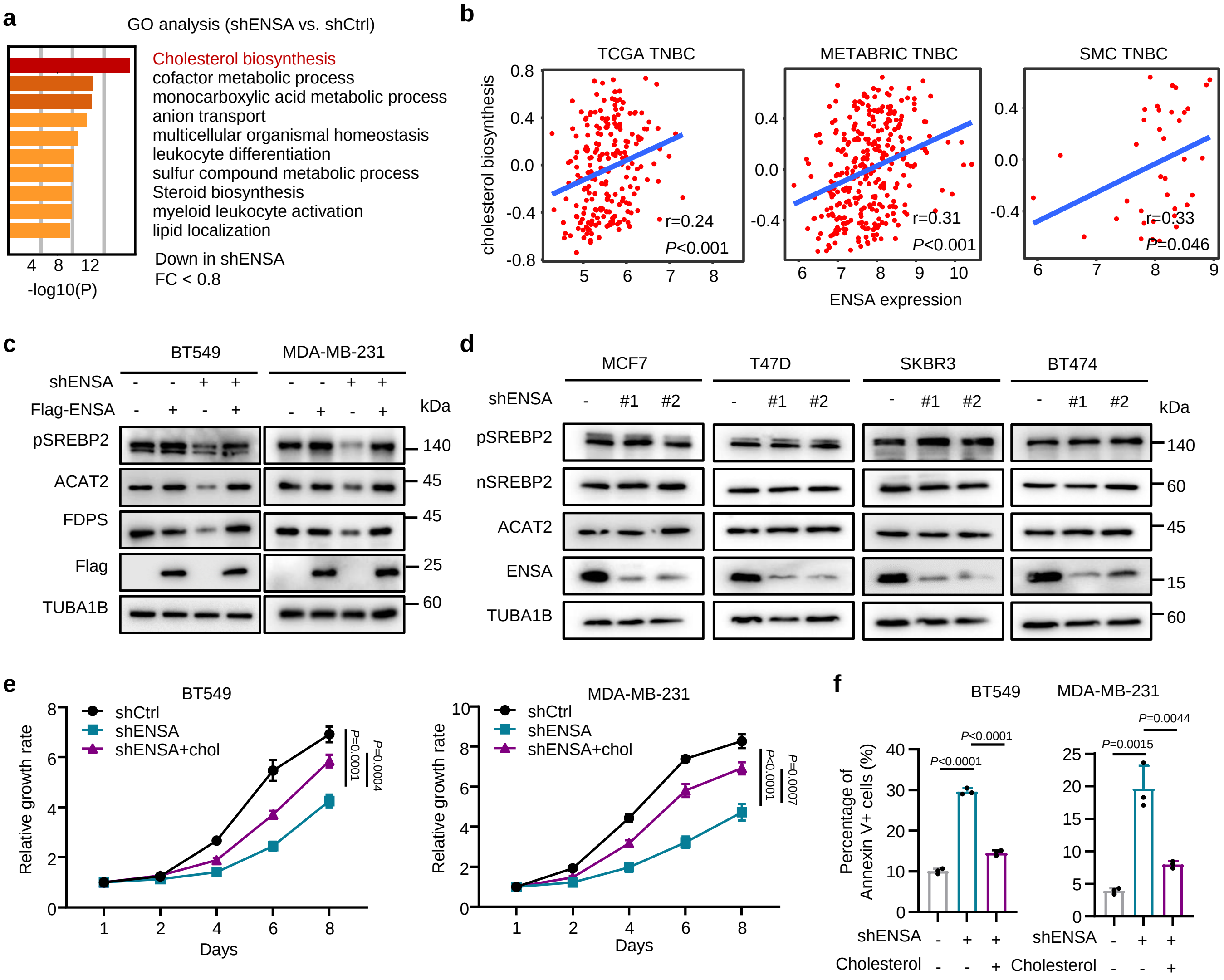
a
GO analysis (shENSA vs. shCtrl)
Cholesterol biosynthesis
cofactor metabolic process
monocarboxylic acid metabolic process
anion transport
multicellular organismal homeostasis
leukocyte differentiation
sulfur compound metabolic process
Steroid biosynthesis
myeloid leukocyte activation
lipid localization
Down in shENSA
FC < 0.8
-log10(P)
b
TCGA TNBC
METABRIC TNBC
SMC TNBC
cholesterol biosynthesis
r=0.24
P<0.001
r=0.31
P<0.001
r=0.33
P=0.046
ENSA expression
c
BT549
MDA-MB-231
shENSA
Flag-ENSA
kDa
pSREBP2
ACAT2
FDPS
Flag
TUBA1B
d
MCF7
T47D
SKBR3
BT474
shENSA
#1
#2
pSREBP2
nSREBP2
ACAT2
ENSA
TUBA1B
e
BT549
MDA-MB-231
shCtrl
shENSA
shENSA+chol
Relative growth rate
Days
P=0.0001
P=0.0004
P<0.0001
P=0.0007
f
BT549
MDA-MB-231
Percentage of Annexin V+ cells (%)
P<0.0001
P<0.0001
P=0.0015
P=0.0044
shENSA
Cholesterol

Supplementary Fig. 4 ENSA impacts cholesterol biosynthesis in TNBC. (a) GO analysis of downregulated genes after ENSA knockdown in MDA-MB-231 cells. Top 10 pathways according to -log10 (*P* value) are shown. **(b)** Scatter plot showing the correlation of ENSA expression with the cholesterol biosynthesis pathway score in TCGA, METABRIC and SMC TNBC data identified by 'gsva' method. Correlation coefficients were calculated using the *Pearson* test. Two-tailed *P*-values were given. **(c)** Western blotting images showing proteins involved in the cholesterol biosynthesis pathway in BT549 and MDA-MB-231 cells ± ENSA knockdown and ± ENSA overexpression. n=3 independent experiments. **(d)** Western blotting images showing proteins involved in the cholesterol biosynthesis pathway after ENSA knockdown in non-TNBC breast cancer cells. n=3 independent experiments. (**e**) In vitro growth curves of BT549 and MDA-MB-231 cells expressing control or ENSA shRNA after treatment with 2.5 μg/ml exogeneous cholesterol. n=3. Data are presented as mean ± SD. Two-tailed two-way ANOVA tests. (**f**) Cell apoptosis measured in BT549 and MDA-MB-231 cells expressing control or ENSA shRNA after treatment with 2.5 μg/ml exogeneous cholesterol. n=3. Data are presented as mean ± SD. Two-tailed unpaired Student's t tests. Source data are provided as a Source Data file. GO, Gene Ontology; FC, fold change; TCGA, The Cancer Genome Atlas; METABRIC, Molecular Taxonomy of Breast Cancer International Consortium; SMC, a Korean breast cancer cohort; chol, cholesterol.

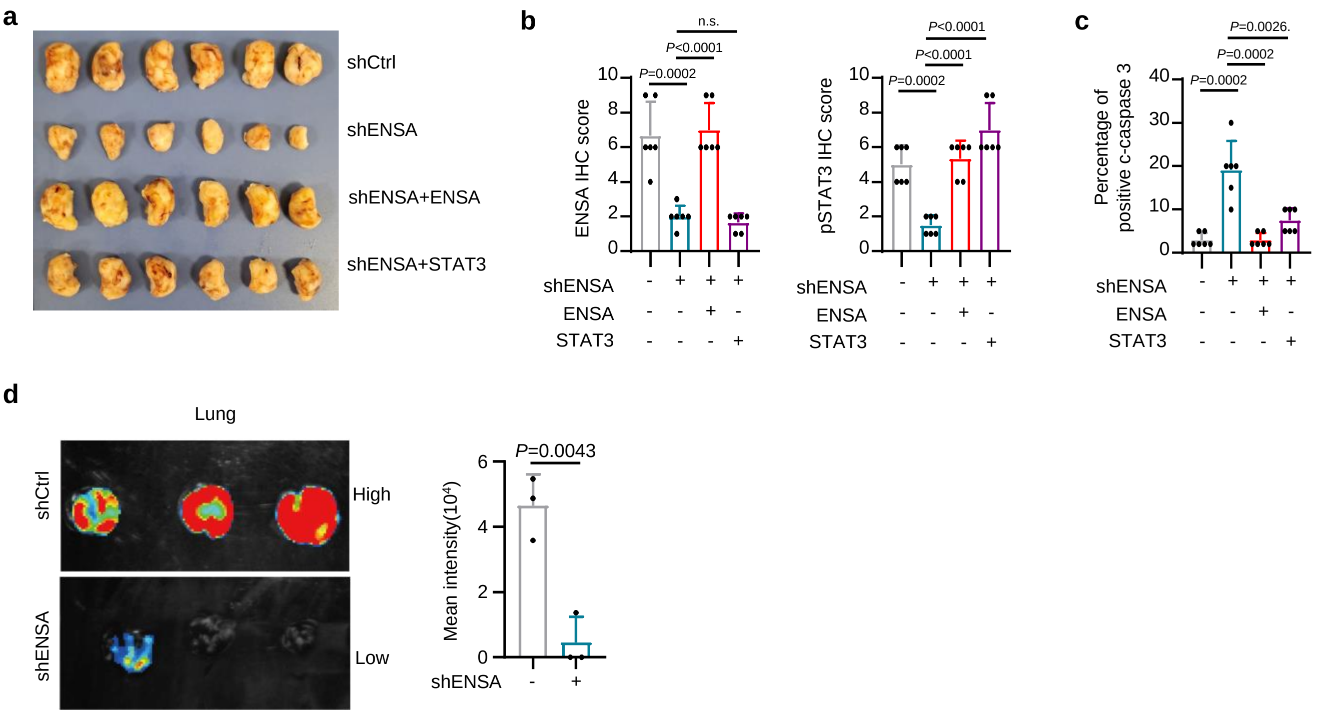

a
shCtrl
shENSA
shENSA+ENSA
shENSA+STAT3
b
ENSA IHC score
n.s.
P<0.0001
P=0.0002
pSTAT3 IHC score
P<0.0001
P<0.0001
P=0.0002
shENSA - + + +
ENSA - - + -
STAT3 - - - +
c
Percentage of positive c-caspase 3
P=0.0026.
P=0.0002
P=0.0002
d
Lung
shCtrl
High
shENSA
Low
Mean intensity(10^4)
P=0.0043
shENSA - +

Supplementary Fig. 5 ENSA/STAT3 promotes TNBC progression in vivo. (a) Representative images of tumors generated by injecting MDA-MB-231 cells expressing control or ENSA shRNA and rescued by ENSA or STAT3 overexpression. **(b)** Immunohistochemical scores of ENSA and pSTAT3-Tyr705 in mouse mammary fat pad xenograft models generated by injecting MDA-MB-231 cells expressing control or ENSA shRNA and rescued by ENSA or STAT3 overexpression. n=6. Data are presented as mean ± SD. Two-tailed unpaired Student's t tests. **(c)** Percentage of cleaved caspase 3-positive cells in mouse mammary fat pad xenograft models. n=6. Data are presented as mean ± SD. Two-tailed unpaired Student's t tests. **(d)** The bioluminescence intensities of lung metastasis of MDA-MB-231 cells expressing control or ENSA shRNA. n=3. Data are presented as mean ± SD. Two-tailed unpaired Student's t tests. Source data are provided as a Source Data file. c-caspase 3, cleaved caspase 3. n.s., not significant.

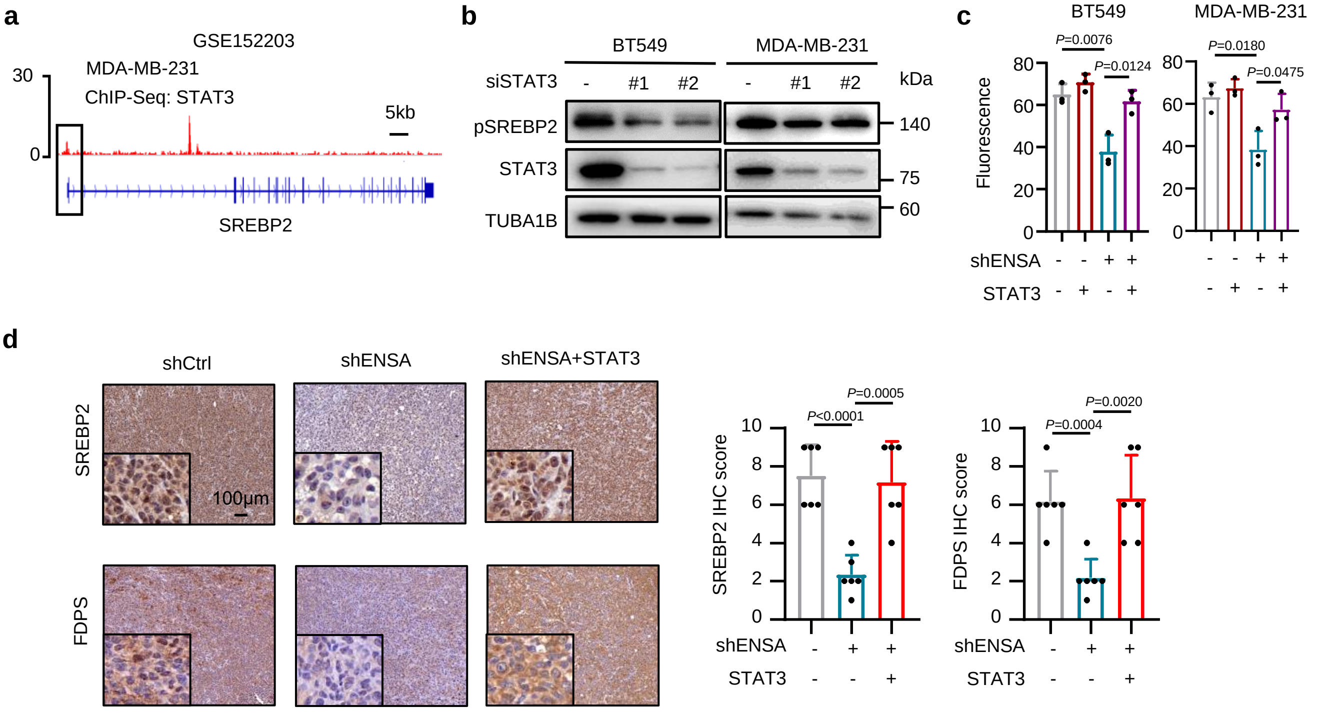
a
GSE152203
MDA-MB-231
ChIP-Seq: STAT3
30
0
5kb
SREBP2
b
BT549
MDA-MB-231
siSTAT3
-
#1
#2
kDa
pSREBP2
STAT3
TUBA1B
140
75
60
c
BT549
MDA-MB-231
Fluorescence
P=0.0076
P=0.0124
P=0.0180
P=0.0475
shENSA
STAT3
d
shCtrl
shENSA
shENSA+STAT3
SREBP2
FDPS
100μm
SREBP2 IHC score
FDPS IHC score
P<0.0001
P=0.0005
P=0.0004
P=0.0020

Supplementary Fig. 6 ENSA mediates the transcriptional activation of SREBP2 via PP2A-STAT3. (a) STAT3 ChIP-seq signal in MDA-MB-231 cells at the genomic location adjacent to the SREBP2 promoter from GSE152203. **(b)** Western blotting images showing SREBP2 expression in BT549 and MDA-MB-231 cells expressing control or STAT3 siRNA. **(c)** Fluorescence quantification of Filipin staining. n=3. Data are presented as mean ± SD. Two-tailed unpaired Student's t tests. **(d)** Immunohistochemical images and scores of SREBP2 and FDPS in mouse mammary fat pad xenograft models generated by injecting MDA-MB-231 cells expressing control or ENSA shRNA and rescued by STAT3 overexpression. n=6. Data are presented as mean ± SD. Two-tailed unpaired Student's t tests. Source data are provided as a Source Data file. IHC, immunohistochemistry.

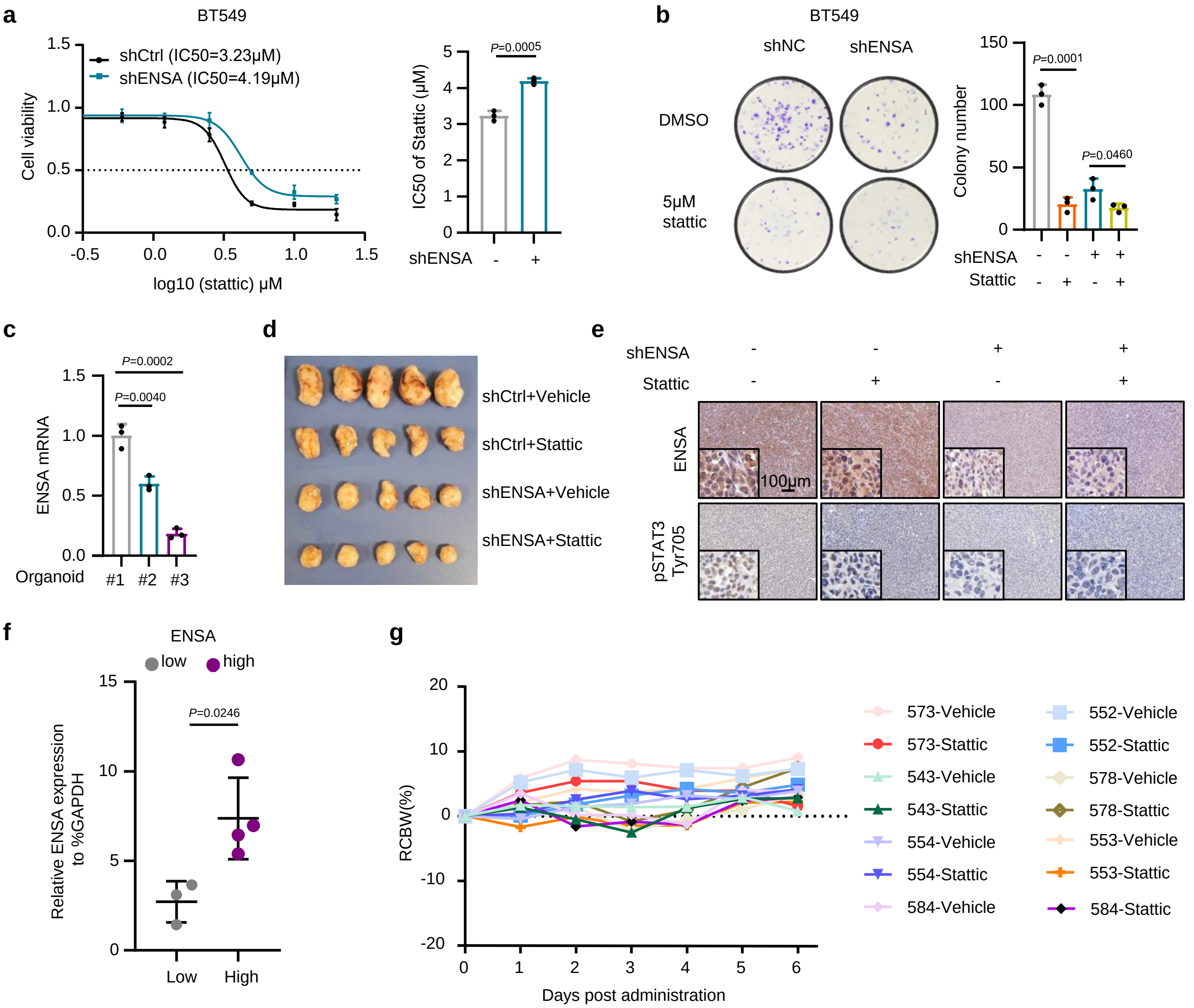
a
BT549
shCtrl (IC50=3.23μM)
shENSA (IC50=4.19μM)
Cell viability
log10 (stattic) μM
P=0.0005
IC50 of Stattic (μM)
shENSA - +
b
BT549
shNC
shENSA
DMSO
5μM stattic
Colony number
P=0.0001
P=0.0460
shENSA - - + +
Stattic - + - +
c
P=0.0002
P=0.0040
ENSA mRNA
Organoid #1 #2 #3
d
shCtrl+Vehicle
shCtrl+Stattic
shENSA+Vehicle
shENSA+Stattic
e
shENSA - - + +
Stattic - + - +
ENSA
100μm
pSTAT3 Tyr705
f
ENSA
low
high
P=0.0246
Relative ENSA expression to %GAPDH
Low
High
g
RCBW(%)
Days post administration
573-Vehicle
573-Stattic
543-Vehicle
543-Stattic
554-Vehicle
554-Stattic
584-Vehicle
552-Vehicle
552-Stattic
578-Vehicle
578-Stattic
553-Vehicle
553-Stattic
584-Stattic

Supplementary Fig. 7 ENSA determines sensitivity to Stattic in TNBC. (a) Dose-response curves and half maximal inhibition concentration values of stattic in BT549 cells expressing control or ENSA shRNA. Dose-response curves: n=6; Data are presented as mean ± SD. Bar plot: n=3 independent experiments; Data are presented as mean ± SD; Two-tailed unpaired Student's t test. **(b)** Clonogenic survival assays of BT549 cells expressing control or ENSA shRNA and treated with 5 μM stattic. n=3. Data are presented as mean ± SD. Two-tailed unpaired Student's t test. **(c)** qRT-PCR detecting relative ENSA mRNA expression in TNBC organoids. n=3. Data are presented as mean ± SD. Two-tailed unpaired Student's t test. **(d)** Representative images of tumors generated by injecting MDA-MB-231 cells expressing control or ENSA shRNA and treating with vehicle or Stattic (10 mg/kg). **(e)** Immunohistochemical images of ENSA and pSTAT3-Tyr705 in mouse mammary fat pad xenograft models. n=3 independent experiments. **(f)** qRT-PCR detecting relative ENSA mRNA expression in TNBC mini-PDX models. n=3 in low-ENSA group and n=4 in high-ENSA group. Data are presented as mean ± SD. Two-tailed unpaired Student's t test. **(g)** Relative body weight changes of mini-PDX-bearing mice. Source data are provided as a Source Data file. RCBW, relative change of body weight.

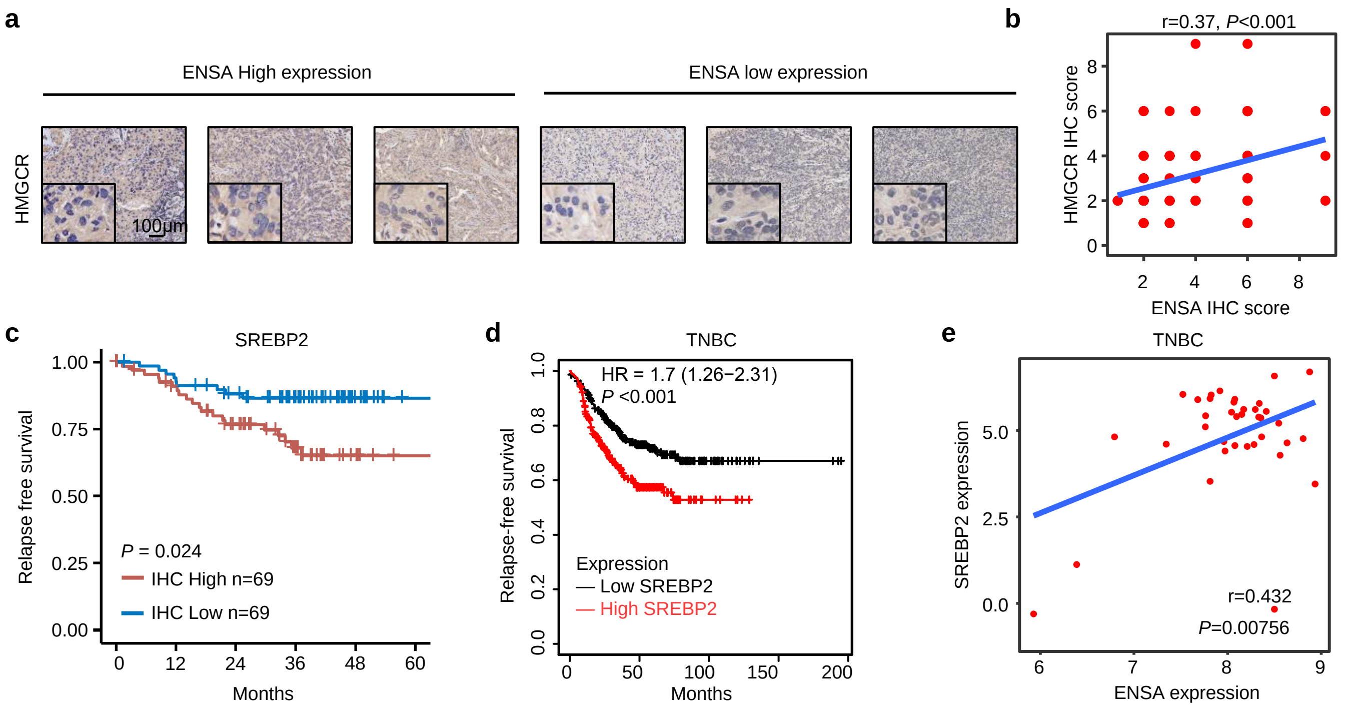

a
ENSA High expression
ENSA low expression
HMGCR
100μm
b
r=0.37, P<0.001
HMGCR IHC score
ENSA IHC score
c
SREBP2
Relapse free survival
P = 0.024
IHC High n=69
IHC Low n=69
Months
d
TNBC
HR = 1.7 (1.26−2.31)
P <0.001
Relapse-free survival
Expression
— Low SREBP2
— High SREBP2
Months
e
TNBC
SREBP2 expression
r=0.432
P=0.00756
ENSA expression

Supplementary Fig. 8 Correlation of ENSA and downstream pathways. (a) Representative IHC images of HMGCR staining in 138 TNBC specimens. Representative images are shown. Scale bars, 100 μm. **(b)** Correlation analysis of ENSA and HMGCR expression levels in 138 TNBC tissues. Correlation coefficients were calculated using the *Spearman* test. Two-tailed *P*-values were given. **(c)** Kaplan-Meier analysis of SREBP2 IHC expression on the relapse-free survival of 138 TNBC patients. Log-rank test. **(d)** Kaplan-Meier plots of SREBP2 in TNBC (https://kmplot.com/analysis/). Log-rank test. **(e)** Correlation analysis of ENSA and SREBP2 expression in an Asian breast cancer cohort (SMC cohort, Korea). n=37 TNBC patients. Correlation coefficients were calculated using the *Pearson* test. Two-tailed *P*-values were given. IHC, immunohistochemistry.

Supplementary Fig. 9 Representative image of FACS sequential gating/sorting strategies of MDA-MB-231 depicted in Supplementary Fig. 3d.

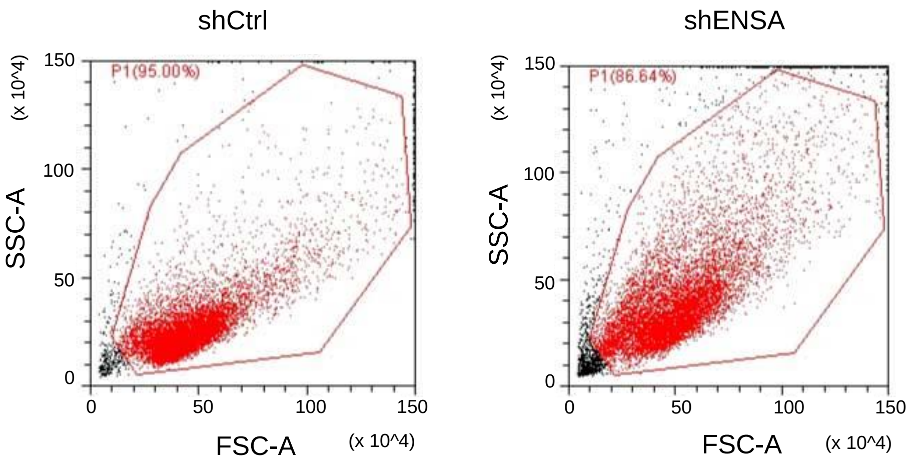

Supplementary Fig. 10 Representative image of FACS sequential gating/sorting strategies of MDA-MB-231 depicted in Supplementary Fig. 3e.

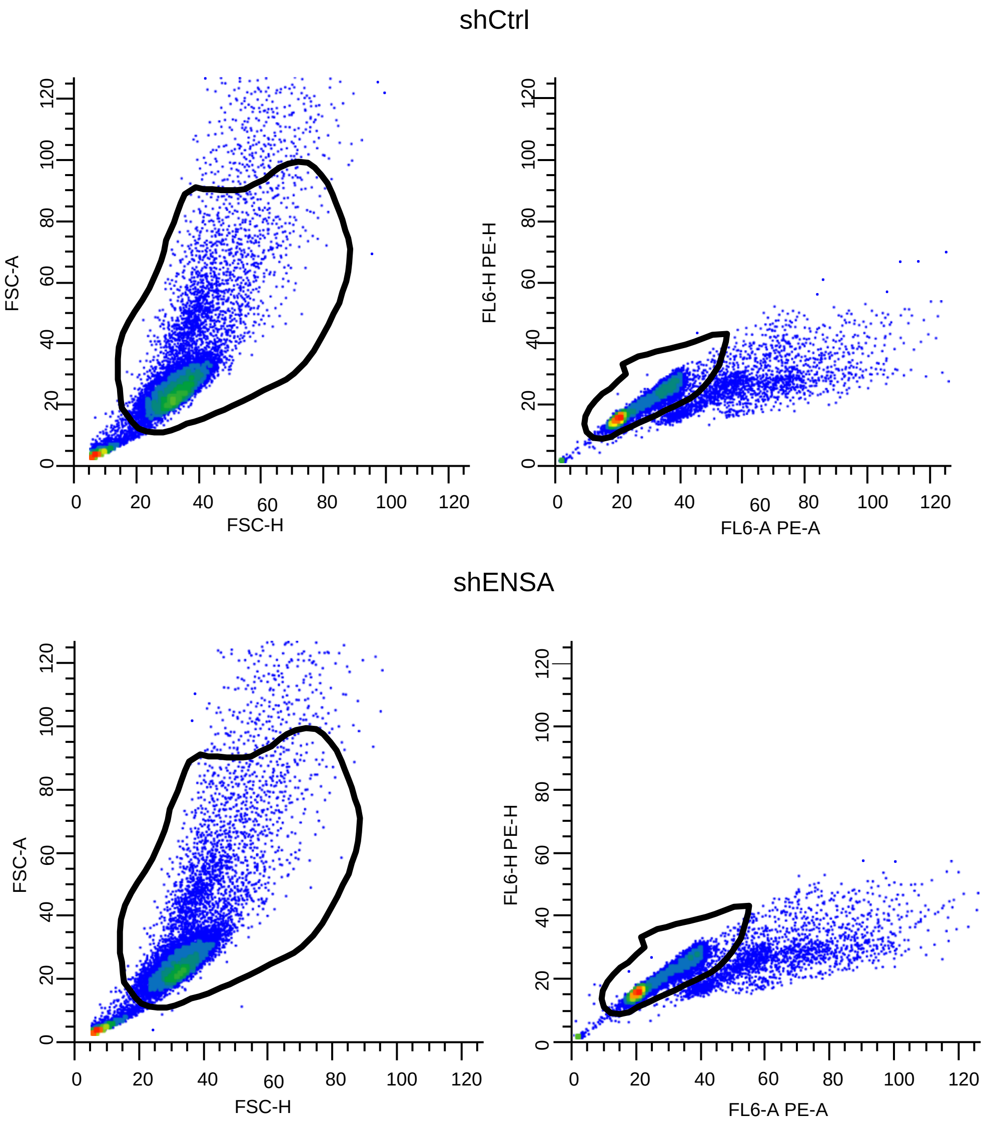

Supplementary Fig. 11 Representative image of FACS sequential gating/sorting strategies of MDA-MB-231 depicted in Supplementary Fig. 4f.

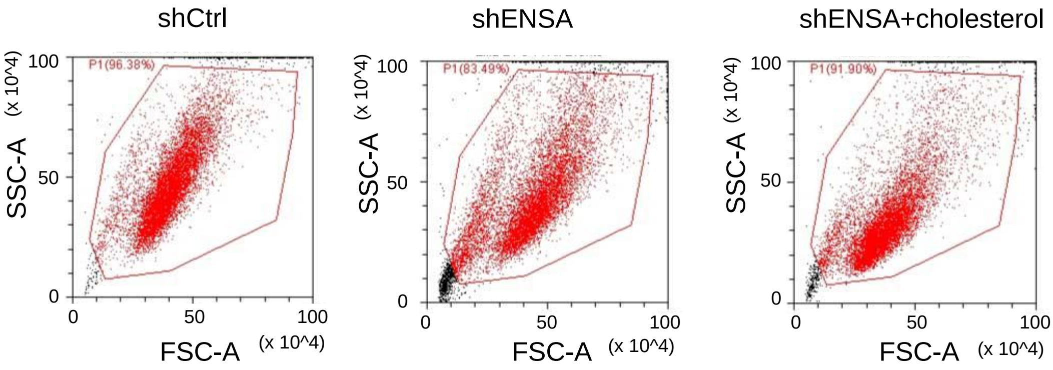

Supplement: Supplementary file 1 — Supplementary Information [file 41467_2022_28452_MOESM1_ESM.pdf]
