# Supplementary material for: Copy number amplification of ENSA promotes the progression of triple-negative breast cancer via cholesterol biosynthesis
Source: Nat Commun. 2022 Feb 10;13:791. doi: 10.1038/s41467-022-28452-z (PMC8831589; doi:10.1038/s41467-022-28452-z)

Immunoblot images depicted in Fig.2a

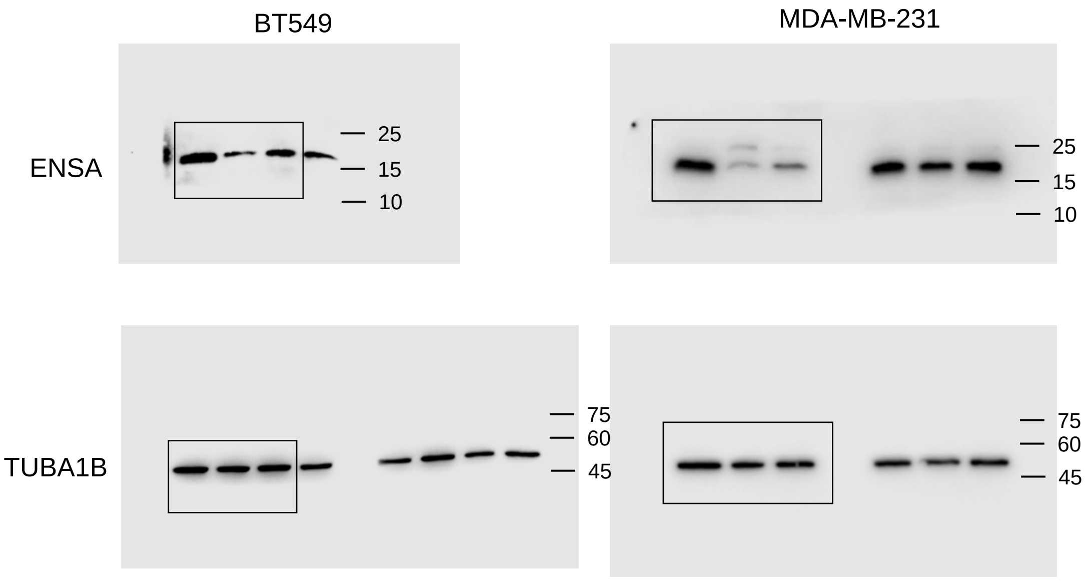

Immunoblot images depicted in Fig.3f

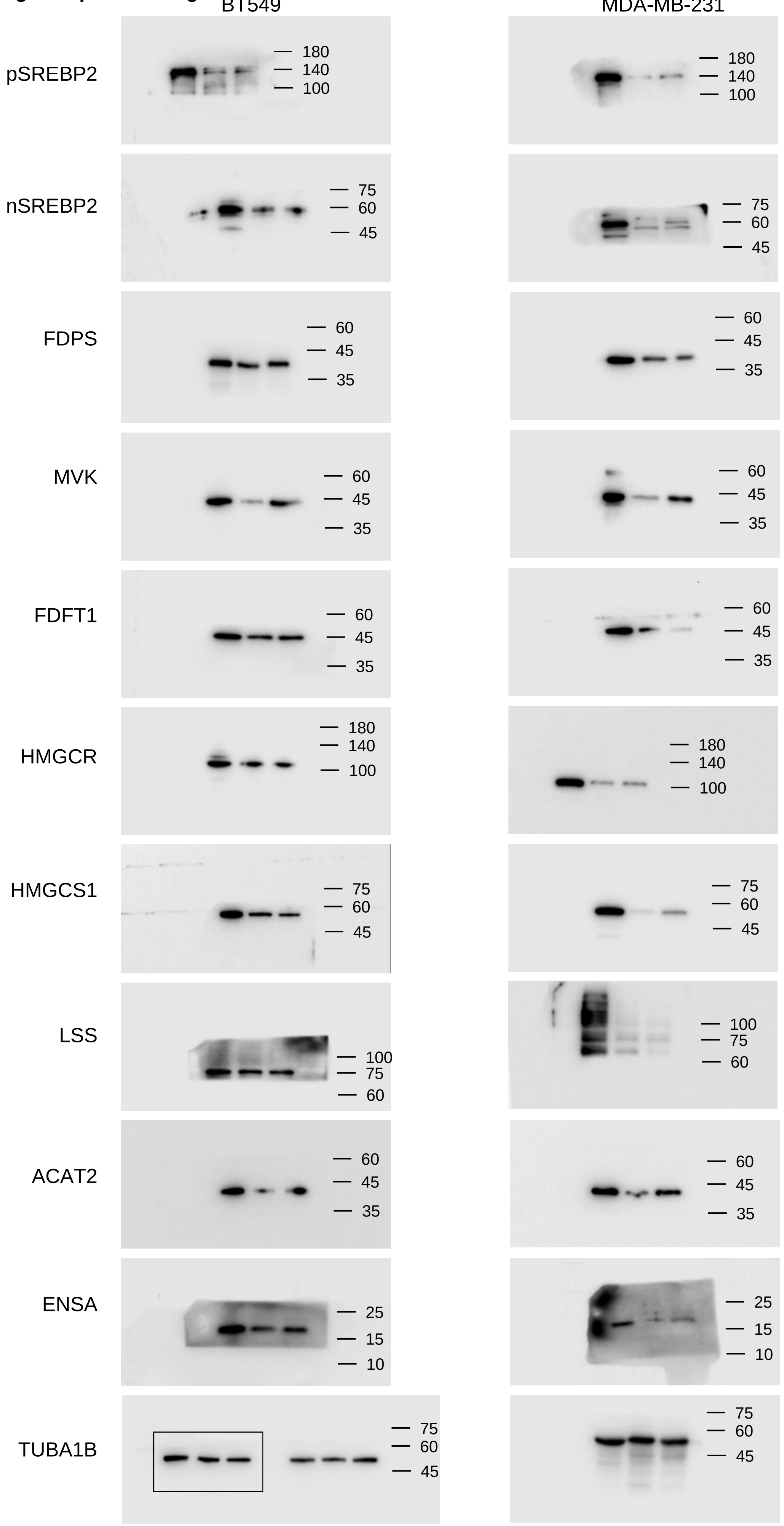

Immunoblot images depicted in Fig.4b

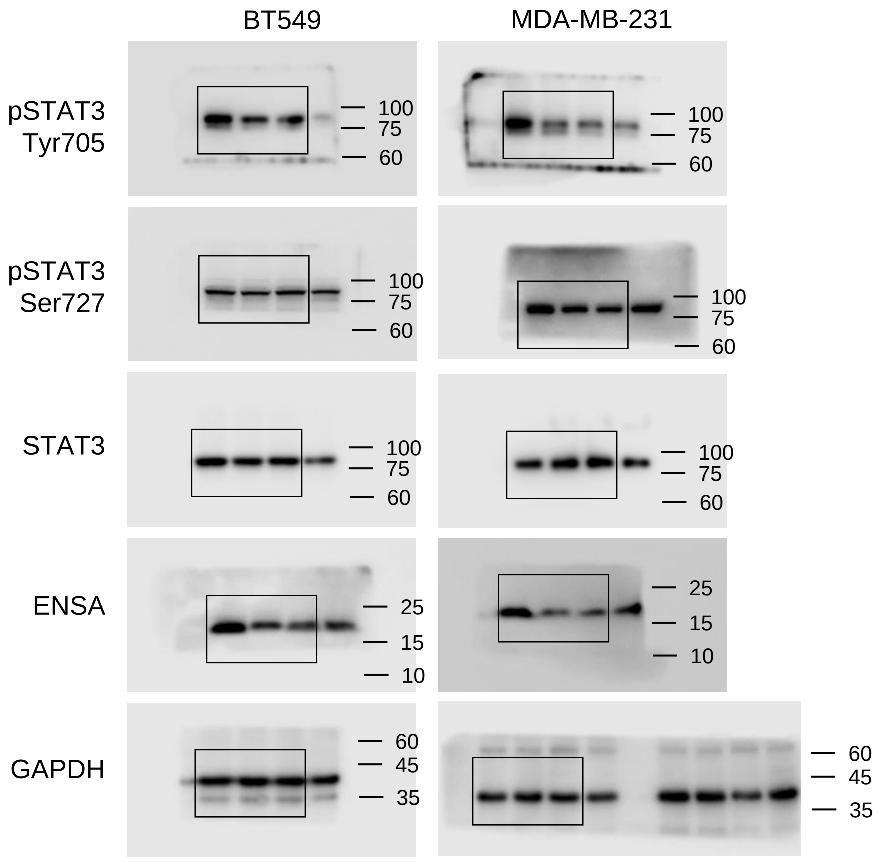

Immunoblot images depicted in Fig.4c

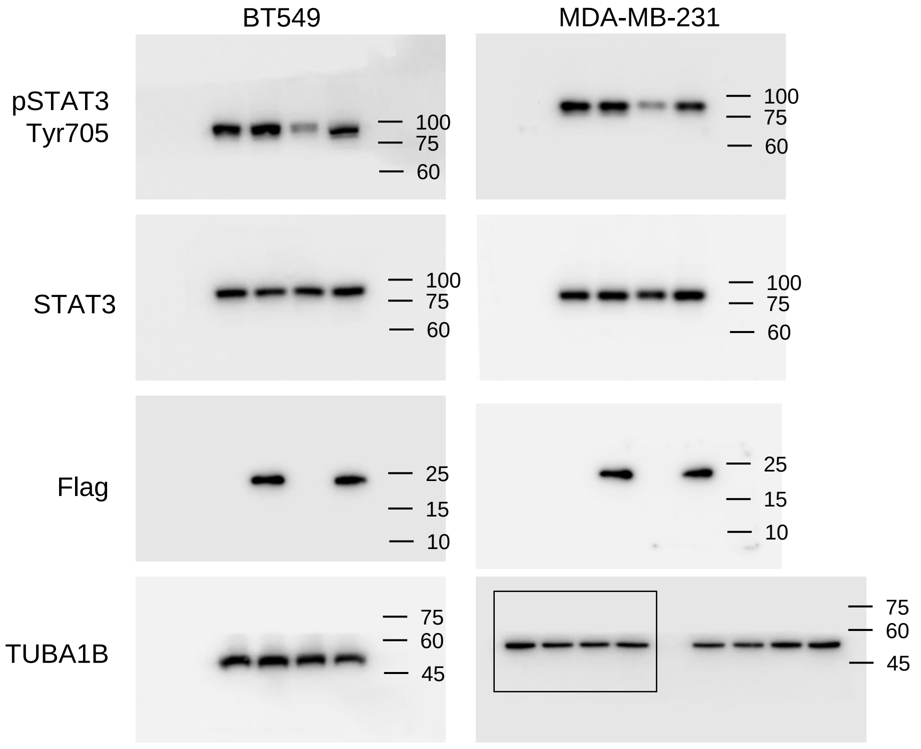

Immunoblot images depicted in Fig.5e

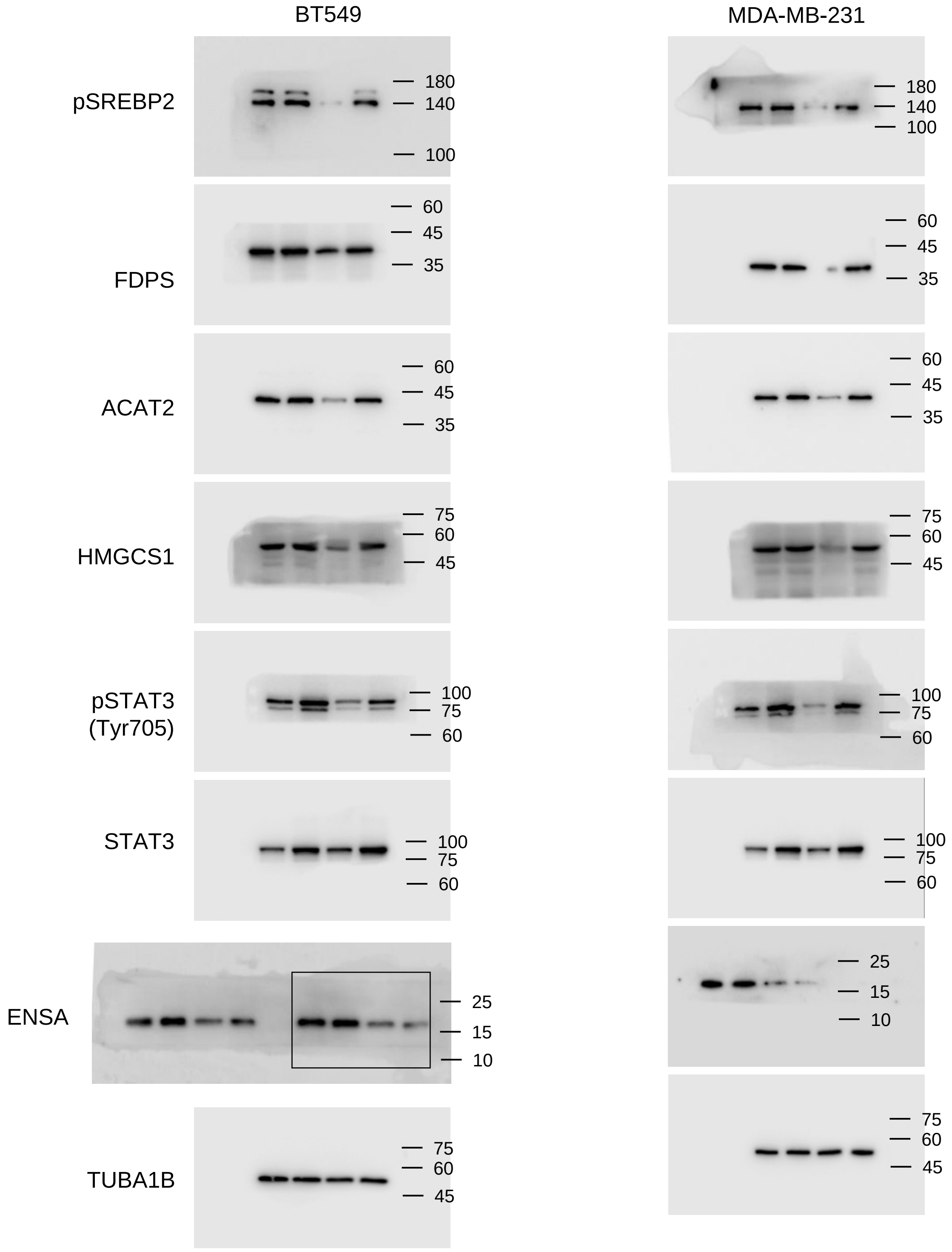

Immunoblot images depicted in Fig.5g

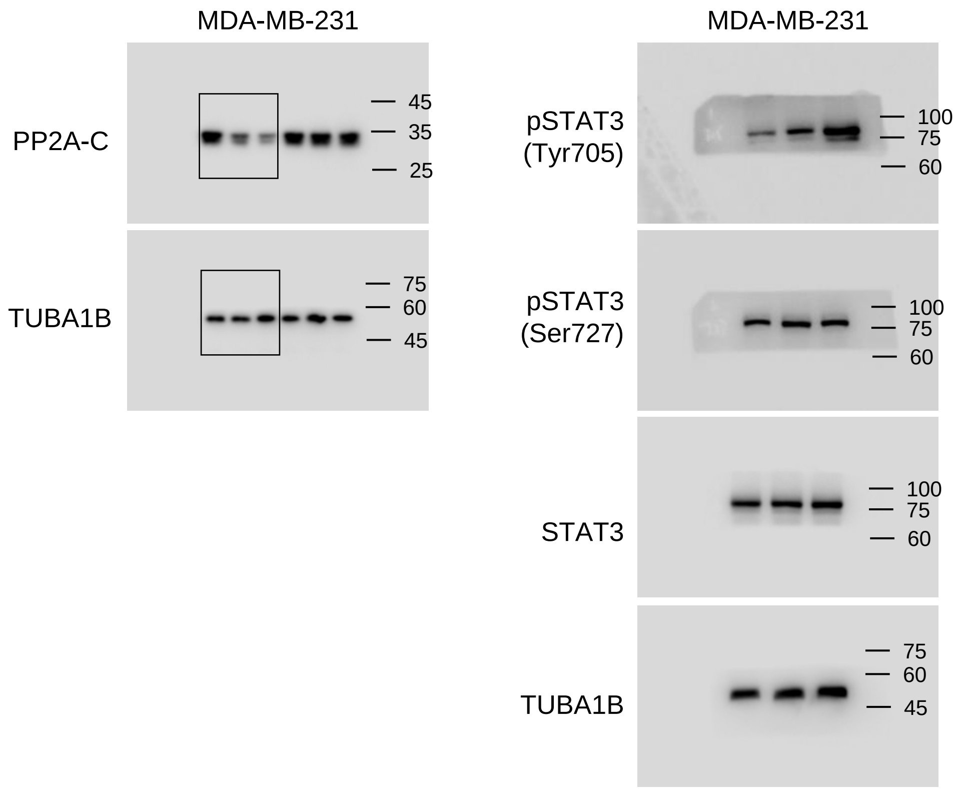

Immunoblot images depicted in Fig.5h

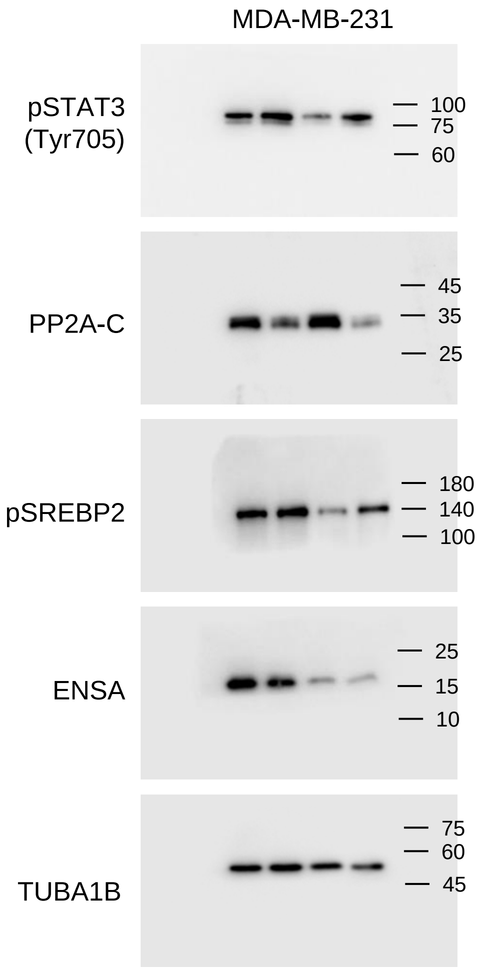

Immunoblot images depicted in Fig.6c

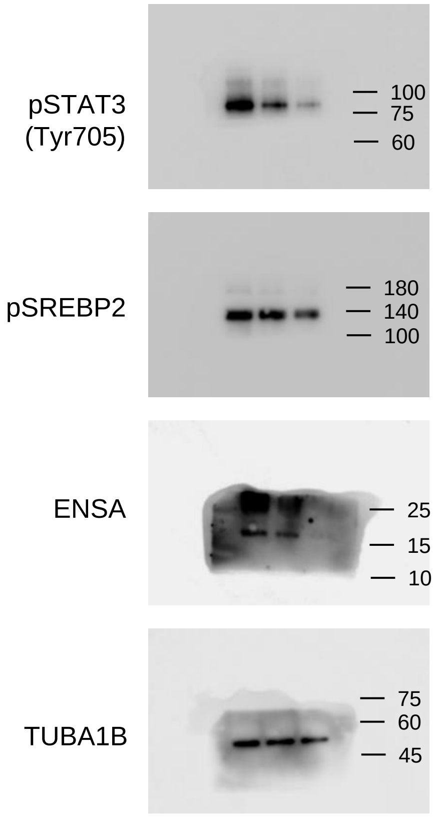

Immunoblot images depicted in Supplementary Fig. 3a

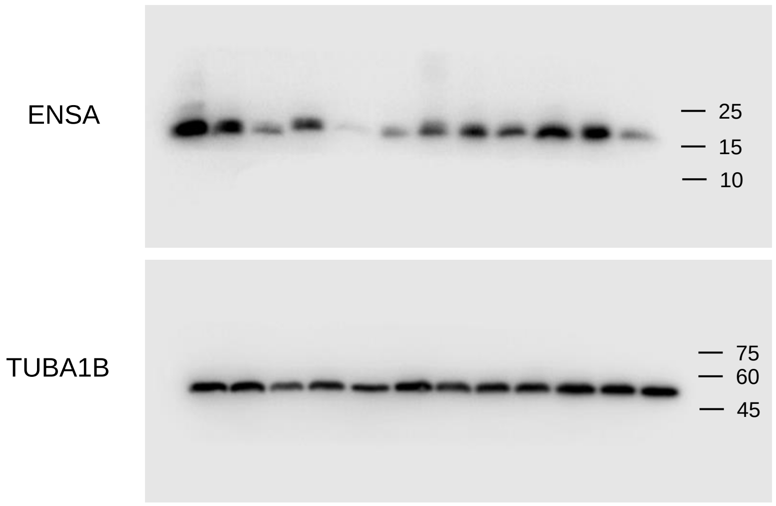

Immunoblot images depicted in Supplementary Fig. 3c

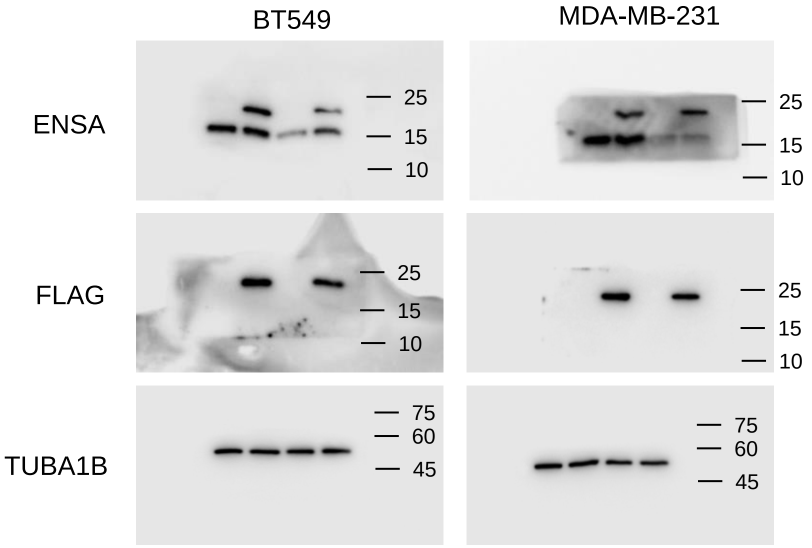

Immunoblot images depicted in Supplementary Fig. 4c

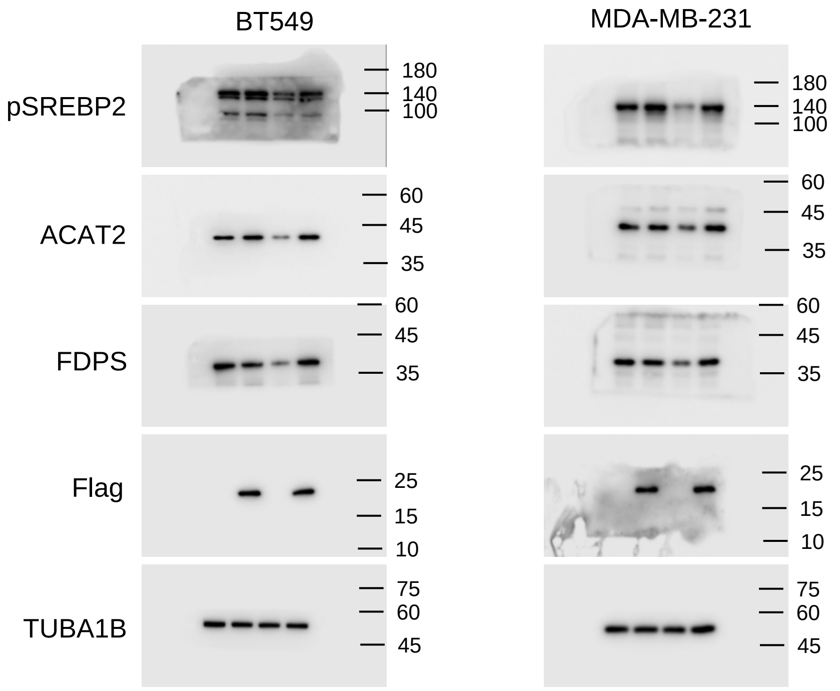

Immunoblot images depicted in Supplementary Fig. 4d

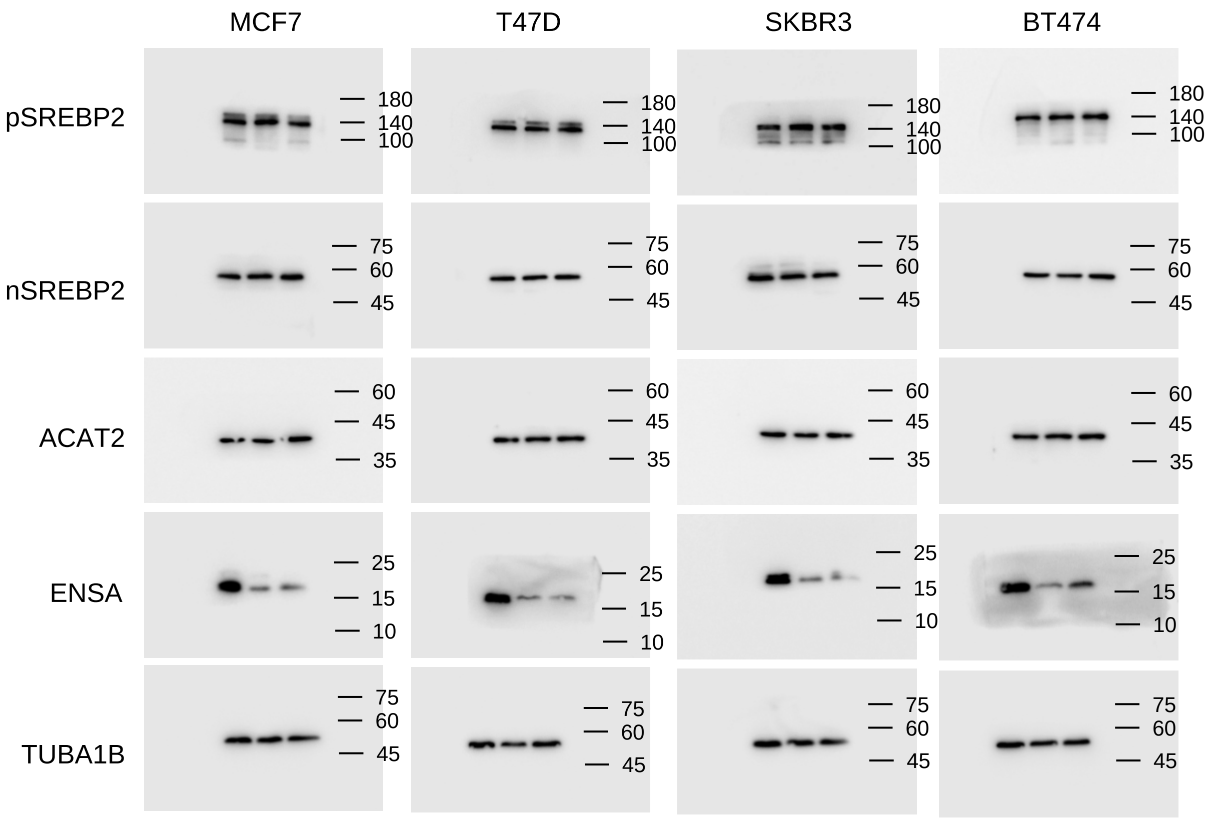

Immunoblot images depicted in Supplementary Fig. 6b

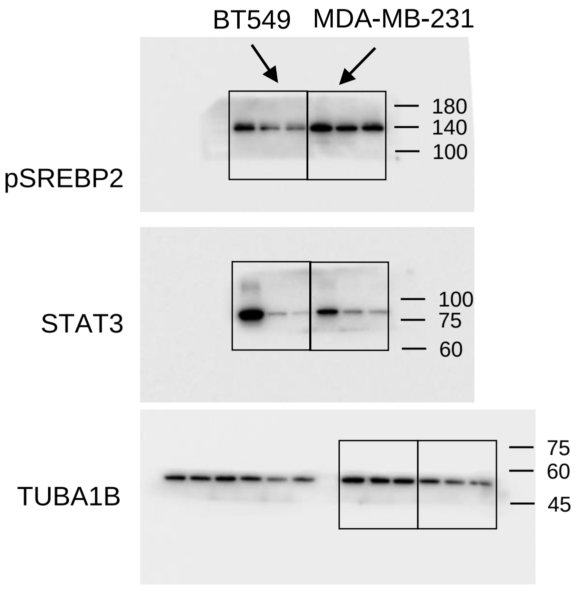

Supplement: Supplementary file 4 — Source Data [file 41467_2022_28452_MOESM4_ESM.zip › Uncropped gel images.pdf]
